# Pharmacy Students’ Experience of an Inaugural Lecture on Intercultural Competence †

**DOI:** 10.3390/pharmacy13050122

**Published:** 2025-09-01

**Authors:** Atta Abbas Naqvi, Merhawi Samsom, Lucy Watson, Hung Nguyen

**Affiliations:** 1 School of Pharmacy, University of Reading, Reading RG6 6UR, UK; 2 International Study and Language Institute, University of Reading, Reading RG6 6UR, UK; 3 Department of Kinesiology and Health Sciences, University of Waterloo, Kitchener, ON N2L 3G1, Canada

**Keywords:** cultural competence, lecture, education, pharmacy, students, pharmacy

## Abstract

**Background:** Pharmacy schools in the United Kingdom (UK) are required by the regulator to train pharmacy students to be culturally competent. To meet this requirement, the Reading School of Pharmacy (RSoP) incorporated an inaugural, stand-alone, introductory session on intercultural competency. This study aimed to gather students’ experiences of the lecture. **Methods:** A qualitative study documented the experiences of students in Years 2 and 3 of the Master of Pharmacy (MPharm) at the RSoP from 15 September to 31 December 2023. Semi-structured interviews were conducted online via Microsoft Teams^®^. A demographic form was prepared and sent as an online survey link on the Online Surveys^®^ platform. All eligible students were invited to participate in the study via student mailing lists. An interview guide was prepared. Thematic analysis was conducted to identify key themes related to students’ awareness, the perceived importance of the subject in healthcare, and students’ preferred learning methods. The transcripts were coded, and similar codes were grouped to form sub-themes and themes. The study was approved by a research ethics committee. **Results:** A total of 11 students attended the interviews. Three major themes emerged: (1) awareness of and reflection on cultural competence, (2) understanding cultural competence and its importance, and (3) student-preferred pedagogy. The students suggested incorporating workshops and simulation-based assessments. **Conclusions:** MPharm pharmacy students at the RSoP appear to be receptive to new educational interventions aimed at enhancing cultural competence. They prefer practice-based learning and assessment methods when it comes to developing this skill.

## 1. Introduction

The UK is becoming increasingly culturally diverse and now represents a population with wide-ranging cultural, ethnic, and linguistic backgrounds. More than 90 languages other than English are spoken in the UK [1]. According to the Office for National Statistics, there are five major ethnic population groups: Asians, Blacks, people of Mixed Race, Whites, and others [1,2]. The most commonly spoken languages after English are Polish, Romanian, Punjabi, Urdu, Portuguese, Spanish, and Arabic, to name a few [1]. A multicultural society means that healthcare professionals will encounter diverse patients and service users. As a result, today’s pharmacy students must be prepared to provide care to individuals from diverse ethnic groups and develop cultural competence when interacting with them. This places an important responsibility on their shoulders to address the needs of a diverse population.

Cultural competence has been defined in various ways in the literature [2]. Cross and colleagues conceptualised cultural competence as a combination of behaviours, attitudes, and policies that, when applied collectively in an organisation or among professionals, enable effective working in cross-cultural settings [3]. Campinha-Bacote conceptualised cultural competence as a continuous process through which healthcare professionals try to work effectively with individuals from different backgrounds [4]. The literature suggests that culturally competent care improves health outcomes, increases patient satisfaction, and reduces health disparities [5,6,7]. In education, there has been an increased interest in designing programmes that meet the needs of students in an increasingly globalised world. The inclusion of Global Citizenship Education (GCE) in the United Nations (UN) Sustainable Development Goals (SDGs) (Target 4.7) has meant that interest in the study and practice of GCE has grown [8]. Many universities now explicitly refer to global citizenship in their education strategy and their curriculum framework; for example, amongst its key ‘graduate attributes’ the University of Reading lists the importance of ‘intercultural competence and international engagement’ [9].

To develop a culturally competent pharmacy workforce for tomorrow, pharmacy education programmes must integrate cultural competence knowledge and adapt assessment to be more culturally inclusive [2]. This need is recognised in the programme learning outcomes outlined by the General Pharmaceutical Council (GPhC), the regulator for pharmacy education and training in England, Wales, and Scotland. The GPhC emphasises the importance of understanding and respecting cultural differences in the MPharm programme [10,11]. To meet this requirement, the Reading School of Pharmacy (RSoP) incorporated a session on intercultural competency. An inaugural lecture on intercultural competency was delivered to pharmacy students, and this study aimed to gather their experiences of the lecture.

It is worth noting that gathering students’ experiences of an educational session helps academics improve the session for better learning and engagement. Students are more likely to engage in a session if their opinions are valued [12]. Collecting feedback also highlights the effectiveness of the lecture and identifies its strengths and weaknesses [13], providing an opportunity to refine the lecture’s contents in the future. Documenting students’ experiences of a session also helps academics understand how students receive new information and identify best practices [14,15]. In addition, it provides insights into how relevant students perceive the new knowledge to be in real-world settings [13]. This is especially important in a practice-based programme such as pharmacy. The aim was to document students’ experience of an inaugural intercultural competency (ICC) lecture delivered at the RSoP. The focus was on assessing how the lecture enhanced their understanding of the topic and how it could be tailored to better meet their needs going forward.

## 2. Methods

### 2.1. Design, Venue, and Eligibility Criteria

This qualitative study involved interviewing pharmacy students studying in Years 2 and 3 at the RSoP. Students who were currently enrolled, had attended the lecture in person, or had watched the recording were eligible to participate. Those who had taken a break from their studies or were unable to progress were not eligible. The interviews were semi-structured and utilised an inductive approach [16]. This approach was chosen to gain a deeper understanding of participants’ perspectives on the lecture. We utilised iterative coding, without pre-conceived ideas, and constantly compared the data to develop categories. Once the coding process had led to the development of categories that had meaning, similar categories were recognised as constituting a theme and were grouped with other themes to form a major theme.

### 2.2. Research Instruments

The research instruments included an online demographic form and an interview guide. The demographic form collected information on participants’ age, gender identity, student status, year of study, ethnicity, and experience of living and working outside the UK.

The interview guide was semi-structured and contained four sections with a total of 13 questions, including prompts. It was designed to encourage open and expansive responses. Section 1 consisted of four questions that assessed participants’ understanding of the importance of cultural competence in both their education and personal life. Section 2 had three questions that identified gaps in pharmacy practice related to cultural competence education and gathered recommendations based on personal experiences. Section 3 also had three questions and captured participants’ feelings, attitudes, and perceptions regarding the ICC lecture. Finally, Section 4 contained three questions that explored participants’ views on the educational materials and assessment methods for cultural competence in the MPharm programme. The interview guide was pilot-tested through a mock interview with a person to ensure its clarity, relevance, appropriateness, and logical flow. The pilot data were not included in the analysis.

### 2.3. Sampling Data Collection Process

The selected sampling strategy was probability sampling, as all participants who fulfilled the eligibility criteria were invited to participate. The data collection process began on 15 September 2023, with an invitation email sent to the participants via student mailing lists. The study invitation email included a weblink directing participants to an online web form on the Online Surveys^®^ platform. The online study link provided access to the participant information sheet, followed by a consent form and a demographic form. Those interested in participating in the interview could provide their contact details through the demographic form. They were then contacted via email to schedule an interview at a mutually convenient time. The data collection period concluded on 31 December 2023.

Interviews were conducted via Microsoft Teams^®^ version 7.14.1, and lasted between 10 min and 35 min. The interviews were conducted one-on-one, with only the investigator and the participant present. The interview was conducted by a student researcher (MS) who had been trained in interviewing and analysing interview data. There was no direct relationship between the interviewer and participants; however, since both were students at the same venue, it is important to acknowledge the possibility of an indirect relationship. The interview guide was also provided to the participants in a follow-up email at the time of scheduling the interview. This allowed the participants to review the questions beforehand and prepare their responses. All interviews were audio–video-recorded. After the interview, a transcript of the meeting was automatically generated by MS Teams^®^. This Teams^®^-generated transcript was then rechecked by researchers against the recorded video to ensure accuracy and rectify any errors. Later, the anonymised transcript was emailed to participants for review.

The interview enrolment continued until data saturation was achieved, which was defined as the point at which an interview no longer produced any significant change in the code book or produced any new information. This approach was guided by the work of Guest and colleagues [17,18]. The study achieved near-saturation by the eighth interview, but an additional three interviews were carried out to confirm the data saturation. The code book is available as Supplementary File S1.

### 2.4. Description of the Lecture

A one-hour lecture, which was part of two compulsory modules, was delivered to the students at the RSoP (N~200) in March 2023 [19,20]. The RSoP offers a Master of Pharmacy (MPharm) degree. It was established in 2004 at the University of Reading Whiteknights campus in Reading [21]. The MPharm programme is a four-year pharmacy degree that teaches pharmacy subjects and allows pharmacy students to attempt a pharmacy registration exam conducted by the regulator, the General Pharmaceutical Council (GPhC), to register to practice as a pharmacist in England, Wales, and Scotland [22]. The instructors were from the RSoP and the International Study and Language Institute, a school that specialises in support for international and foundation students but also delivers general, university-wide optional modules on topics such as global citizenship and intercultural competence, and communication at the University of Reading. The intended learning outcomes of the lecture were to define cultural competence and explain its importance and relevance in healthcare and pharmacy practice.

### 2.5. Data Analysis

The demographic data were analysed using IBM SPSS^®^ version 27 statistics software, and the sample count (*N*) and frequency (%) were reported. There were no missing data. A thematic analysis was conducted to identify underlying themes within the interview data. The data were coded, with each code supported by multiple anonymised quotes from the participants. The quotes were identified by a serial number (P1, P2, etc.). Similar codes were grouped into sub-themes, which were then categorised to highlight major themes. Two researchers independently coded the data. The coding process was driven by the interview data and was not dependent on the interview questions. This preserved the inductive nature of the thematic analysis. Triangulation was conducted by rechecking the thematic analysis individually and through group discussion.

### 2.6. Ethical Clearance

The study was approved by the School Research Ethics Committee (SREC) at the RSoP, University of Reading (approval #42/2023), on 22 August 2023. The survey was anonymous and voluntary, without any pressure to participate. Participants could complete the web form at their convenience, including outside academic premises. They had the option to withdraw from the study. Additionally, they could choose not to answer any question if they found it distressing. The participants were assured of the confidentiality of the interview to encourage open and honest responses. Moreover, the participants were invited to provide confidential feedback to another investigator about the interviewer, which was mostly positive and constructive. The recording and email conversation were deleted once the transcript had been finalised. The participants were reimbursed for their participation. The reporting of qualitative findings adhered to the consolidated criteria for reporting qualitative research (COREQ) guidelines [23].

## 3. Results

A total of 11 students attended the interviews (Table 1). Most participants identified as females (*N* = 7, 63.6%) and belonged to a Black ethnic background (*N* = 3, 27.3%). Some mentioned other ethnic backgrounds, such as Middle Eastern (*N* = 4, 36.4%). Most students were from Year 3 (*N* = 7, 63.6%) and attended the lecture in person (*N* = 10, 90.9%). There was a good distribution when it came to the participants’ student status, as slightly more than half were international students (*N* = 6, 54.5%). A few participants had lived (*N* = 4, 36.4%) and worked (*N* = 2, 18.2%) outside the UK (Table 1). The raw data is available as Supplementary File S2. 

The qualitative analysis produced three themes from the data: awareness of and reflection on cultural competence, understanding cultural competence and its importance, and student-preferred pedagogy. All three themes were major and stand-alone themes that aligned with the aims of the study. All three themes had sub-themes, and each sub-theme had codes that were supported by quotes from the participants. The quotes were anonymised. Each sub-theme provided in-depth details about the viewpoints of the participants. All the themes are reported in the order of the theme, sub-theme, and code. The analysis is also supported by selected anonymised quotes from the participants.

### 3.1. Theme 1: Awareness of and Reflection on Cultural Competence

**Figure 1:**
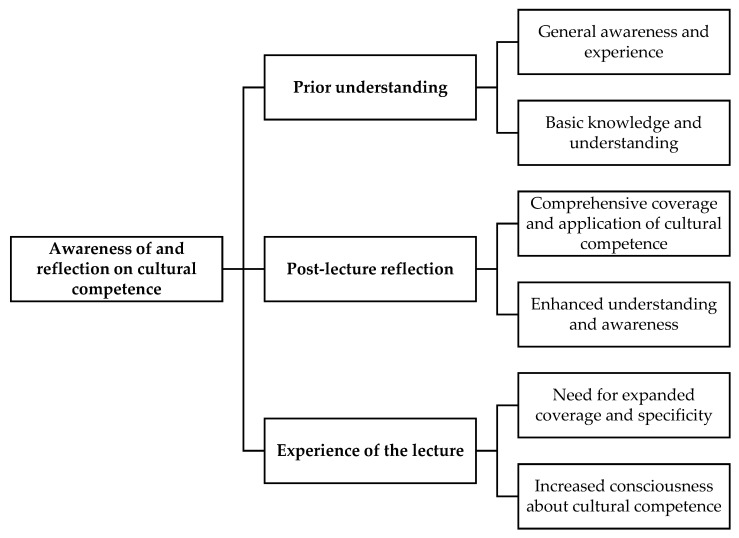
Theme diagram for awareness of and reflection on cultural competence.

The students shared their knowledge, awareness, and experiences before and after engaging in the educational session on cultural competence. They described their pre-existing understanding and the subsequent improvement in their knowledge and highlighted the significance of learning cultural competence in their practice (Figure 1).

#### 3.1.1. Prior Understanding

Students shared that they had an understanding of the topic and shared their pre-existing knowledge, awareness, and personal experience of cultural competence. They had a general awareness of cultural differences and believed that their behaviour needed to be somewhat aligned with the beliefs of people from other cultures.

*I can say generally, like to know people coming from different cultures and our behaviour or towards them should be little bit aligned with what they believe*.

(P2)

Some had previous exposure to cross-cultural knowledge and/or engaged with people from diverse cultures.

*I am from a different country like I’m not originally from the UK, I feel like I had very general grasp on the concept because I would understand that people might not know my culture, so I would like be understandable, if they don’t, but I will feel really appreciated if they do*.

(P7)

Others did not have a deeper understanding.

*I didn’t have that much understanding about it. I just knew that I thought it is just like respecting to other people cultures and how you deal with different ideas, different point, and uh opinions of people that was as my idea for going to the lecture*.

(P6)

*I had basic experience with it for my own professional life. But I didn’t have deeper understanding*.

(P10)

#### 3.1.2. Post-Lecture Reflection

The students reflected on their experience after the lecture and shared that there was an improvement in their understanding of cultural competence, awareness of biases, and application of CC skills in practice. They mentioned that they had an improved comprehension of the topic and greater self-recognition of biases and respect for other cultures, and were able to relate this to their practice in the future.

*The topic for me meant being aware of my own cultural beliefs and values, and how that differed from people in different communities. It included being able to participate and honour a variety of different cultures. I think the lecture did a great job at educating me on the biases and the stereotypes we can feed into and how that affects the care that we give to patients, and this was something I was unaware of*.

(P3)

*I had a bit more understanding of how to approach different situations post-lecture*.

(P4)

*I think this lecture brought something better and more valuable for me as a future healthcare professional*.

(P9)

The students felt that the lecture was able to cover the topic of cultural competence and were able to see how this skill affects the care they need to provide to their clients in the future as healthcare professionals.

*I found the lecture being very covering through the whole concept*.

(P7)

*Like different people have different beliefs, culture, backgrounds and as of probably as a pharmacist, you try not to judge that person based on what they think. You just try to understand and try to help them. Even though they have different background, try to help them. Regarding the background and show that they’re not different, they’re just like everyone, and they will get the help they needed no matter what their background is*.

(P6)

*I got a greater number of examples of where it can be used in practice, like how to look out for it and be more aware*.

(P8)

#### 3.1.3. Experience of Lecture

The participants shared their experiences after the lecture and expressed a desire for more specialised materials on cultural competence and the integration of more practice-based scenarios that highlight the application of cultural competence skills.

*The topic is very wide and very important, I think they should make more content on that and then on different lectures, because I would say 1 lecture isn’t enough to cover all of it*.

(P9)

*It had enough details to teach us what it is, what intercultural competency is. But it didn’t go beyond any further teaching, so more detail could be better*.

(P10)

*I suppose it could go into more specific details*.

(P11)

Some asked for more information on specific topics within cultural competence.

*The topic of emotional intelligence. how to improve your emotional intelligence when you’re dealing with patients from different backgrounds and making sure you’re being professional and being culturally competent*.

(P10)

*I don’t know if this counts like but for mute and blind people like that they cannot communicate. I don’t think it counts in cultural competence, but I feel like because he talked about people who have difficulty in communicating, I feel like general education about mute and blind people will be something to add*.

(P7)

The students also shared that the lecture had increased their consciousness about cultural competence and they now realised the importance of understanding and respecting persons from different cultures.

*The way to understand other people’s culture and relate that to like your own experience So not everybody might share the same like cultural background, but it’s important to know about others*.

(P1)

*I believe that it changes my understanding by firstly making me more conscious about the important of different culture that I might encounter*.

(P11)

*I think I definitely thought more about cultural competence than I have before. So that was good. I think it’s an important conversation*.

(P4)

### 3.2. Theme 2: Understanding Cultural Competence and Its Importance

**Figure 2:**
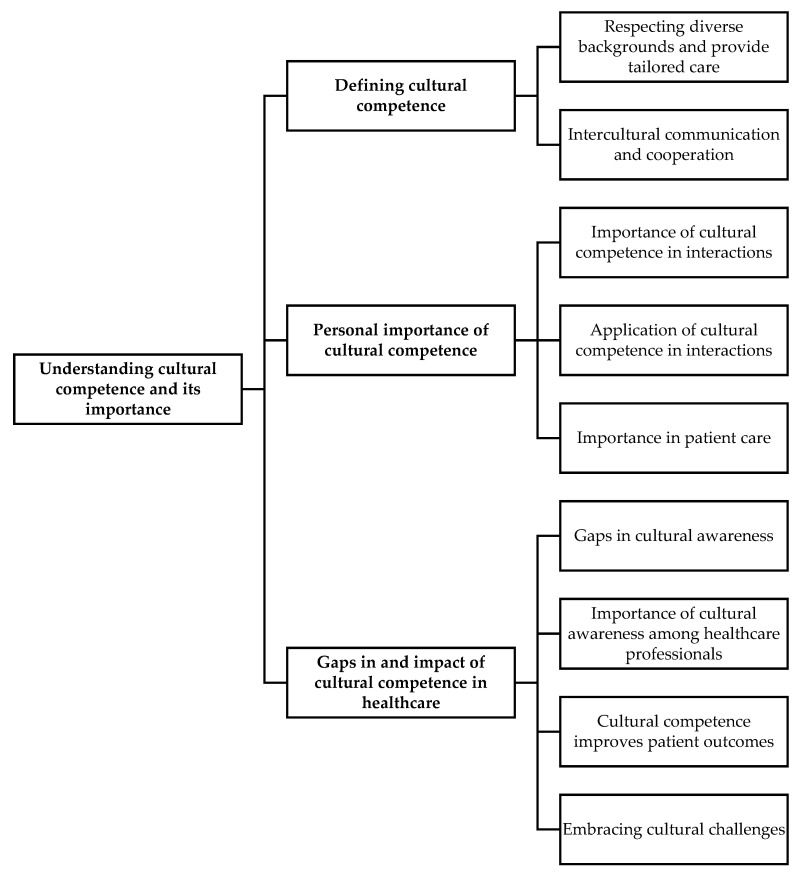
Theme diagram for understanding cultural competence and its importance.

This theme included recognising the importance of cultural competence skills such as cultural awareness, knowledge, sensitivity, etc., in providing an effective healthcare service and in achieving positive patient outcomes. This theme included the definition of cultural competence from the participants’ perspective, the role it plays in the personal performance of a healthcare professional in their role, and the need to enhance their intercultural competency skills and eliminate biases by engaging in continuing professional development (Figure 2).

#### 3.2.1. Defining Cultural Competence

The participants shared their own version of the definition of cultural competence, which centred around recognition of and respect for diverse backgrounds, elimination of biases during care, and tailoring communication according to the person’s background and culture.

*Cultural competence to me, it means recognising that other people are different to yourself, and they have different backgrounds, different identities, and being able to, uh, provide care for them regardless of their background in an equal manner*.

(P10)

*You are able to understand the different cultures and respect different cultures. That you have the enough knowledge and confidence to act and provide services, talk to people from different cultures without doing any harms as specialized healthcare professional*.

(P11)

*There are some people with different backgrounds and then you have to respect uh and then deal with their backgrounds and their beliefs and respect their opinions*.

(P9)

The students mentioned that intercultural communication and cooperation with persons from different cultures is also at the core of cultural competence.

*Intercultural competence, just from the name sounds to me how well you can, uh, cooperate and communicate with different people from different cultures. It within your workplace or within educational institutions*.

(P11)

*I think it’s just to implement that different people have different opinions based off of their cultures and you should be able to, you know, work with people with these different backgrounds despite your different opinions*.

(P11)

#### 3.2.2. Personal Importance of Cultural Competence

This sub-theme highlighted students’ perspectives about their realisation of the importance of cultural awareness and sensitivity, and respecting and empathising with persons during diverse interactions. It also included addressing cultural differences and language issues that might hinder the achievement of person-centred care. The students mentioned the importance of being culturally aware and understanding, and respecting the different backgrounds of individuals when interacting with them either in the capacity of a pharmacist or in their personal life. This also included the ability to understand and empathise with people from different backgrounds and cultures, and being able to recognise and respect diverse perspectives during an interaction.

*So just like being able to understand how other people’s culture like affects your interactions with them and like how important, like how embedded culture is*.

(P1)

*I think it’s important to have the ability to understand and interact effectively with people from different cultures. It allows us to have self-awareness, have empathy towards one another, and the chance to create long lasting relationship*.

(P3)

*It is important to be aware of other people’s ethnic, backgrounds, like not cause offence to them and like accidentally insulting them or something. But I think it’s important for both Pharmacy and in just in life in general*.

(P8)

The participants also mentioned their tips on how to be culturally competent during interactions. Application of cultural competence skills meant being culturally aware and sensitive when interacting with persons from different backgrounds. This further included addressing language barriers and cultural differences in healthcare service delivery.

*I would say just make sure you know the language. Make sure you’re empathetic and understanding, no matter what, and don’t try and undermine someone’s health condition just because maybe their English is not so good because you might be like missing it important red flag symptoms*.

(P10)

Others mentioned the need to deal with a problem in a cross-cultural setting in a professional manner. However, they could not explain specifics.

*Probably maybe like how to deal with it. Like If you face any problems in the future look like in a cultural difference or requires a cultural competence, and like how to actually handle it or deal with it as like in a professional way, as a pharmacist, how would we deal with it in the future. if you actually face that in and that in a work setting*.

(P6)

The participants also recognised the role of cultural awareness and sensitivity in patient care and how it impacts achievement of person-centred care.

*Maybe just be like a little bit more cautious about what you say in front of patients. Just be like cautious what you say to different patients. different patients may react differently to information that you give them*.

(P8)

*If you lack intercultural competence, so if you [are] lacking intercultural competence, you could damage a patient’s health and for that reason it’s more important in pharmacy practice*.

(P10)

*When it comes to pharmacy practice, it’s just having a cultural competence is important because it helps you to achieve that patient centred care much better and better quality*.

(P11)

#### 3.2.3. Gaps in and Impact of Cultural Competence in Healthcare

The students shared that they felt that there are gaps in cultural awareness among most members of the public and some healthcare professionals. They felt that there was an unfamiliarity with diverse cultures among most members of the public.

*I don’t see many people kind of eager to find out about other people’s culture in a sense*.

(P1)

*Most of the people here at least, or at least I can say the people that I’ve been in contact with, or people that I’ve seen. They are not familiar with all the cultures apart from just some common cultures that you can see in the UK*.

(P2)

*Some other healthcare professionals such as pharmacy dispenses and are rarely aware of the importance of intercultural competence. As I have witnessed in some situation where pharmacy dispenser gets confused, if patient behaved differently and because of a religion or their culture beliefs and they were expecting him or her to behave as normal, but that wasn’t the case and then they were confused*.

(P9)

They emphasised the need for healthcare professionals to possess necessary skills to deliver effective healthcare and achieve positive patient outcomes.

*I think that every pharmacist has to have a good knowledge of different religions and cultures. Because I think it makes the patient more able to communicate and then more open to speak up about their diseases or illnesses with health professional for better treatment*.

(P9)

*I think that would kind of like affect a future pharmacist and practice if they’re not eager to find out where their patients are coming from and trying like give a better, like better healthcare towards them because like surrounding their culture, I think that that’s why it’s quite important*.

(P1)

*I think that pharmacist and healthcare professionals in general should familiarise themselves with diverse cultural backgrounds because it will affect the care that they give. This includes different beliefs, different values and healthcare practices, and how this will affect their response to medication and treatment*.

(P3)

They also highlighted how having cultural competence would impact the care provided to persons in a positive manner.

*Being interculturally competent improves patient outcomes because it allows you to communicate better and be more empathetic, which has an impact on the patient’s quality of care*.

(P10)

*We need to have the knowledge, uh of different background of the patient because it helps in offering a patient centred care*.

(P9)

*I think if they try to reach that communication to be able to communicate the feelings or the cultural background, then the pharmacist might get a better understanding of what’s going on*.

(P11)

They further called on healthcare professionals to challenge themselves to address cross-cultural issues in healthcare by engaging in continuing professional development to improve their intercultural competency.

*But this is something I would want to just like try and like step out of your comfort zone*.

(P1)

*I think one of the gaps is being able to receive training for putting aside your different political beliefs. Maybe I might have a different political belief to you, but we still have to be able to provide that same care and we haven’t really been given that training before*.

(P10)

### 3.3. Theme 3: Student-Preferred Pedagogy

**Figure 3:**
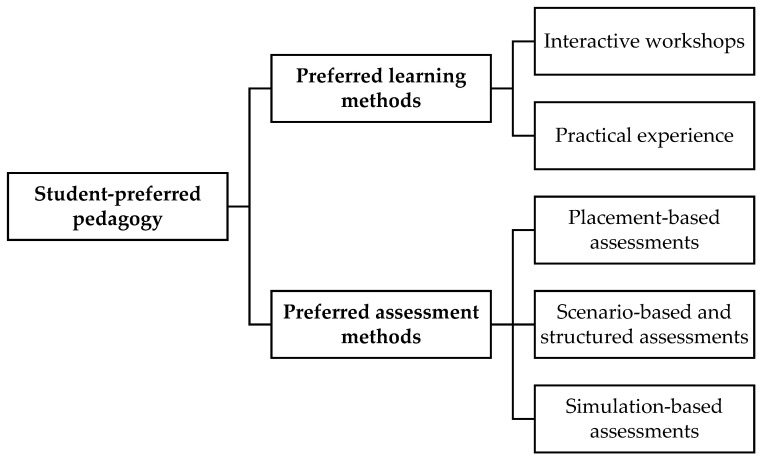
Theme diagram for student-preferred pedagogy.

This theme included the learning and assessment methods that the students preferred for the teaching and assessment of cultural competence in the curriculum. It included the students’ preferences for integrating interactive and engaging learning methods such as workshops and placements and several assessment techniques, such as scenario-based, simulation-based, placement-based, and structured assessments (Figure 3).

#### 3.3.1. Preferred Learning Methods

The participants mentioned their preference for the incorporation of interactive learning methods in the curriculum that enhance engagement and help students acquaint themselves with cross-cultural situations that arise in real-world healthcare settings. The students mentioned having workshops that are interactive and allow for roleplays and discussion to improve their understanding of cross-cultural scenarios and require demonstration of cultural competence skills as part of the solution to a problem.

*In the workshops you could have mock role plays, so you could like someone can be acting as a patient, someone acting as a pharmacist*.

(P10)

*I think I would benefit from workshops that would allow me to build on skills such as like analysing and being able to interpret verbal and nonverbal cues with patients who may not speak English*.

(P3)

*The content if delivered as a lecture won’t be as effective as if it was as a workshop because students could be more interactive with the content, and it could be like scenario based*.

(P4)

Some preferred the integration of direct, hands-on, experiential learning opportunities such as placements that provide exposure to cross-cultural situations in healthcare settings.

*Maybe talk in practice like doing in practical like simulations instead of lecture*.

(P7)

*So, like in placements, it is a good way to actually get direct experience with other patients from different cultures*.

(P10)

#### 3.3.2. Preferred Assessment Methods

This included the myriad methods students would prefer to be assessed using with regard to their intercultural competence skills. These include placement-based, scenario-based, structured, and simulation-based assessments.

Some students preferred having an assessment of their cultural competence skills during an experiential learning activity such as a placement.

*I think intercultural competence is something you have to have in general as a professional, so it’s maybe like part of professional practice. like scenario-based decision making*.

(P4)

*I think it says that placement activity assessment was an example. I think that would be a great way because maybe we can like role play different scenarios with patients of different backgrounds. It allows us to be practical and put ourselves in their shoes*.

(P3)

Others mentioned using specific evaluation techniques and assessment methods to measure a student’s cultural competence skills in an examination. These included multiple-choice questions (MCQs) and scenario-based questions.

*I think it would fit more in the scenario-based questions*.

(P4)

*Culture competence can be assessed as a group study. Group case study like presenting a problem or try to solve the problems*.

(P6)

*Maybe an MCQ such as what would you do in this case? Uh, like more of an ethical problem, I guess it would be, right? Or maybe like short answer question, or you can even make maybe a long answer question. You know, like a situation with a patient like that maybe. So, it’s like making a scenario and see how they will react to it*.

(P5)

Students also mentioned employing mock roleplays and simulation-based assessments such as objective structured clinical examinations (OSCEs) to assess intercultural competency.

*I feel like if it’s more of a simulation thing, like in our OSCE, you can do like ohh, I’m a person of colour or an Asian person and I have different beliefs from you. What do you do then? So that will put you in a difficult position to actually deal with the problems. I feel like it’s a thing for OSCE mostly like put it in the OSCE; don’t think it fits in exam questions*.

(P7)

*I think OSCE might be good for the assessment*.

(P1)

*I think we have OSCE’s ahead of us and we have some practical as well that we talk to the patients. I think the best assessment could be dealing as practice… And the teachers can monitor as if we consider the patient’s culture*.

(P2)

## 4. Discussion

This study explored the perspectives of pharmacy students at the RSoP regarding the inclusion of cultural competence education in the MPharm programme. It examined their views on how this education impacted students’ awareness of cultural competence, their self-perceived importance of the topic, and their preferred learning methods. The findings align with the current efforts by the National Health Service (NHS) [24] and the GPhC to promote culturally competent, inclusive, and person-centred care [10,11].

The students acknowledged that they had some prior understanding of cultural competence, though it was basic and superficial, which was primarily shaped by their personal experiences. This understanding included acknowledging diversity and showing respect to people from different cultures and backgrounds. However, after reflecting on their experience of the lecture, they reported significant improvements in their understanding of the concept, awareness of biases, cultural sensitivity, and ability to apply these skills in practice. This approach is consistent with the current literature, which supports the introduction of a themed lecture earlier in the degree programme to help students familiarise themselves with the concept [25,26].

The students expressed a desire for more content on cultural competence, particularly with a focus on nuanced, practice-based learning. They expressed an interest in exploring more cross-cultural scenarios relevant to practice. Liu and colleagues similarly found that medical students were eager to learn about applying cultural competence to practice [27]. They acknowledged the role of cultural competence in fostering an environment of cultural safety for individuals with diverse needs. Thus, this aligns with the lecture’s objective, which was to encourage students to consider how to address the needs of persons from different backgrounds, a principle embedded in the NHS constitution [28] and the GPhC’s strategy [10,11].

The participants identified gaps in cultural awareness and practice within healthcare. They stressed the need to engage more with people from diverse backgrounds, recognising this as essential for providing quality care. This underscores the importance of further embedding cultural competence in the MPharm programme and expanding the continued professional development opportunities for practicing pharmacy professionals to enhance this skill.

One pedagogical approach to developing pharmacy students’ cultural competence skills during the MPharm is to immerse them in cross-cultural scenarios, increasing their cultural awareness and knowledge. This exposure allows them to observe culturally appropriate practices and their impact on healthcare services and vice versa. The interactions students have with individuals from diverse backgrounds enable them to develop intercultural communication skills. They can then reflect on their cross-cultural observations and encounters to further enhance their skills for the future [29]. The School has integrated placements across all years of the MPharm programme. These placements, arranged in hospitals, general practices, and community pharmacies, aim to enhance students’ clinical skills as well as cultural competence. These placements are designed to meet the learning outcomes specified in the GPhC’s MPharm curriculum framework. However, there is a potential for further research to assess the impact of these placements on the cultural competence of pharmacy students.

The students preferred experiential learning methods such as workshops, roleplays, simulations, and placements, as they believed that these methods are more effective in delivering knowledge. This response aligns with the views of pharmacy students from another higher education institution (HEI) in the UK [26]. Placements have been suggested as a suitable teaching and learning method for developing cultural competence by pharmacy students in another UK pharmacy school [26]. Other studies focusing on nursing have also emphasised the importance of placements, practicums, and clinical experiences in sharpening cultural competence skills. In addition, research in nursing education has highlighted simulation-based teaching as an effective method for enhancing this skill among nursing students [29,30]. A systematic review by Walkowska and colleagues highlighted the positive impact of using simulations to teach cultural competence to healthcare students [31]. Similarly, Jarar and colleagues reported that didactic learning, experiential learning, case-based learning, and simulation are among the most frequently used teaching and learning methods for delivering cultural competence knowledge to pharmacy students [2].

In terms of assessing cultural competence, the participants proposed myriad ways to evaluate this skill. These included placement-based observation and simulated environments such as an objective structured clinical examination (OSCE). Some suggested assessing cultural competence through scenario-based essay questions. The approach of evaluating this skill through practice-based assessment supports the concept of authentic assessments, which aim to measure cultural competence among pharmacy students in a real-world context. In higher education, the authenticity of an assessment refers to how well the assessment replicates real-life situations or, in healthcare, real practice [32]. This finding is reported here for the first time. By incorporating authentic assessments such as placement observations or simulation-based evaluations, educators can more effectively assess students’ cultural competence skills.

This study gathered student responses from a single venue and was based on a single lecture. This significantly limits the transferability of the findings across other higher education institutions (HEIs) in the UK. In addition, the findings relied on students’ self-reporting, which introduced the possibility of social desirability bias. However, despite these limitations, the study provides insights into how pharmacy students feel about including educational sessions on topics such as cultural competence. It can also serve as a guide for academics across HEIs in the UK when designing new sessions on this subject.

## 5. Conclusions

This study highlights several key messages. Firstly, it establishes that pharmacy students recognise the importance of cultural competence skills in their practice and show positive acceptance of such sessions. Secondly, while a stand-alone lecture may be suitable at the start of the programme to introduce key concepts, it may not be sufficient to ensure that students feel confident in applying cultural competence in clinical settings. Further sessions need to be delivered by instructors who also have knowledge and an understanding of cultural competence. Thus, more interactive sessions incorporating roleplay, case-based learning, and simulation-based learning may be necessary in later stages of the programme. The preference for authentic assessments highlights clear pathways for improving the pharmacy curriculum and ultimately boosting students’ confidence in applying cultural competence in practice. 

## Figures and Tables

**Table 1:** Demographic information (*N* = 11).

Variables	*N*	(%)
**Age**		
18–20	4	36.4
21–23	4	36.4
24 and above	3	27.3
**Gender**		
Male	4	36.4
Female	7	63.6
Ethnicity *		
Asian	2	18.2
Black	3	27.3
Mixed	1	9.1
White	1	9.1
Any other ethnic groups	4	36.4
**Year of study**		
Year 2	4	36.4
Year 3	7	63.6
**Lecture engagement**		
Attended in person	10	90.9
Watched recording	1	9.1
**Student status**		
International	6	54.5
Home	5	45.5
**Lived outside the UK for 6 months or more**		
Yes	4	36.4
No	7	63.6
**Worked outside the UK**		
Yes	2	18.2
No	9	81.8

* As per the Census 2021 of England and Wales. Legend: Asian = Asian British, Indian, Pakistani, Bangladeshi, Chinese, Arab, OR any other Asian background. Black = Black British, Caribbean, or African, OR any other Black, Black British, or Caribbean background. Mixed = multiple ethnic groups, White and Black Caribbean, White and Black African, White and Asian, OR any other Mixed or multiple ethnic background. White = White, English, Welsh, Scottish, Northern Irish or British, Irish, Gypsy or Irish Traveller, Roma, OR any other White background.

## Data Availability

The data are available as Supplementary Files.

## Supplementary Materials

The following supporting information can be downloaded at: https://www.mdpi.com/article/10.3390/pharmacy13050122/s1, File S1: Code book; File S2: Raw data.
