# Supplementary material for: Pharmacy Students’ Experience of an Inaugural Lecture on Intercultural Competence
Source: Pharmacy (Basel). 2025 Sep 1;13(5):122. doi: 10.3390/pharmacy13050122 (PMC12452684; doi:10.3390/pharmacy13050122)
Supplement: Supplementary file 1 [file pharmacy-13-00122-s001.zip › File S1. Code book.pdf]

Code book

1. Awareness and reflection on cultural competence [Theme]

This theme covers the knowledge, awareness, and experiences of participants before and after engaging in the educational session on cultural competence. It furthers shows the evolution of the pre-existing understanding and subsequent improvement in participants' knowledge, and their perspectives on the topic. Further, the significance of the topic in practice has been highlighted as well.

1.1. Prior understanding [sub-theme]

This is the pre-existing knowledge, awareness and personal experience a person has regarding cultural competence. This sub-theme includes general awareness, basic knowledge, limitations in understanding and prior experience of a person regarding cultural competence.

1.1.a. General awareness and experience

General awareness is the ability to recognise and understand the difference in cultures and be able to tailor one's own behaviour to align with people from other cultures. Prior experience means that the person had previous exposure to cross cultural knowledge or engaged with people from diverse cultures.

1.1.b. Basic knowledge and understanding

A foundation-level of understanding about cultural competence. It means that there is an acknowledgment of having a superficial knowledge about cultural competence that requires further improvement.

**Codes**	**General Awareness and experience**	**Basic Knowledge and Understanding**
**Quotes**	I can say generally, like to know People coming from different cultures and our behaviour or towards them should be little bit aligned with what they believe. (P2)<br><br>I am from a different country like I'm not originally from the UK, I feel like I had very general graphs on the concept because I would understand	I did have some background knowledge before we had the lecture. However, I just had the basics. (P4)<br><br>I didn't have that much understanding about it. I just knew that I thought it is just like respecting to other people cultures and how you deal with different ideas, different point, and uh opinions of people that was as my idea for going to the lecture.(P6)

	that people might not know my culture, so I would like be understandable, if they don't, but I will feel really appreciated if they do. (P7)<br><br>I had taken some courses similar to it before. So I understand the culture Competence. Different cultures, things related to that. I would say before the lecture, I would say, I'm an international minded person, so I understood it anyway. (P11)	I had basic experience with it for my own professional life. But I didn't have deeper understanding. (P10)

1.2. Post-lecture reflection [sub-theme]

It refers to the responses of participants after the educational engagement. This includes the improvement in understanding of cultural competence, awareness of biases, and application of this skill in practice.

1.2.a. Enhanced understanding and awareness

This refers to the improved comprehension and humility towards persons from different cultures. It also includes recognition of preconceived ideas and beliefs a person has about individuals from different backgrounds and cultures, and the ways in which patient care is impact because of it. It further includes effective approaches in cross-cultural situation learned through an educational experience.

1.2.b. Comprehensive coverage and application of cultural competence

This refers to the extent to which the educational experience covered the topic of cultural competence and highlighted the practical application of cultural competence skills in healthcare practice.

**Codes**	**Enhanced understanding and awareness**	**Comprehensive coverage and application of cultural competency**
**Quotes**	I think the lecture did a great job at educating me on the biases and the stereotypes we can feed into and how that affects the care that we give to patients And this was something I was unaware of. (P3)	There is no other area where you could have had that conversation where it mentions culture competency. (P4)<br><br>I found the lecture being very covering through the whole concept. (P7)

	I had a bit more understanding of how to approach different situations post lecture. (P4)<br><br>I think it was important to have that lecture just to spread our awareness and what we can expect in the future. (P6)<br><br>I think this lecture brought something better and more valuable for me as a future healthcare professional. (P9)<br><br>I think the lecture was holistic, so it gathered all of the core pieces of information that you need for a broad understanding. (P11)<br><br>The topic for me meant being aware of my own cultural beliefs and values, and how that differed from people in different communities. It included being able to participate and honour a variety of different cultures. (P3)	Again, getting to find out more about other people in my course and the importance of cultural competence within pharmacy was part of the good side of the lecture. (P1)<br><br>Like different people have different beliefs, culture, backgrounds and as of probably as a pharmacist, you try not judge that person based on what they think. You just try to understand and try to help them. Even though they have different background try to help them. Regarding the background and show that they're not different, they're just like everyone and they will get the help they needed. no matter what their background is. (P6)<br><br>I got a greater number of examples of where it can be used in practice. like how to look out for it and be more aware. (P8)

1.3. Lecture experience [sub-theme]

The perspective of participants on the educational experience that included their desire for more comprehensive and specialised materials. This also includes the impact of the session on participants' perceptions about its significance.

1.3.a. Need for expanded coverage and specificity

This reflects the desire for more detailed and specialised piece of information on cultural competence. This includes a desire to integrate practice scenarios highlighting the application of cultural competence skills, in the educational curriculum.

1.3.b. Increased consciousness about cultural competence

This means that there is an improved understanding of the significance of cultural competence after the learning experience. This includes the realisation of the need to understand and respect persons from different cultures.

Codes	Need for expanded coverage and specificity	Increased consciousness about cultural competence
Quotes	It had enough details to teach us what it is, what intercultural competency is. But it didn't go beyond any further teaching, so more detail could be better. (P10)<br><br>I think the lecture didn't talk about how cultural competency, how that training is going to take place in pharmacy profession in the following year. I don't know if it's going to be in pre reg or if we going to have workshop about it throughout the year. (P3)<br><br>I suppose it could go into more specific details. (P11)<br><br>I would appreciate like maybe more discussion. (P1)<br><br>The topic of emotional intelligence. how to improve your emotional intelligence when you're dealing with patients from different backgrounds and making sure you're being professional and being culturally competent. (P10)<br><br>I don't know if this counts like but for mute and blind people like that they cannot communicate. I don't think it counts in cultural competence, but I feel like because he talked about people who have difficulty in communicating, I feel like general education about mute and blind people will be something to add. (P7)<br><br>the topic is very wide and very important, I think they should make more content on that and then on different lectures, because I would say 1 lecture isn't enough to cover all of it. (P9)<br><br>I think culture is not just religion. (P2)<br><br>I think if we add an audit during the placement might be beneficial for students as well. (P9)	I believe that it changes my understanding by firstly making me more conscious about the important of different culture that I might encounter. (P11)<br><br>The way to understand other people's culture and relate that to like your own experience So not everybody might share the same like cultural background, but it's important to know about others. (P1)<br><br>I would say like I saw more of it, like it's importance within like the pharmacy field. (P1)<br><br>I think I definitely thought more about cultural competence than I have before. So that was good. I think it's an important conversation. (P4)<br><br>I did understand some stuff. Like we are all different and we must know about the different religions and cultures because when they come to your pharmacy, you won't ask them or what culture are you and you need to think and recognize them basically. (P5)

2. Understanding cultural competence and its importance [Theme]

This theme includes recognising the importance of cultural competence skills such as cultural awareness, knowledge, sensitivity, etc., in providing effective healthcare service and achieving patient outcomes. This theme includes definition of cultural competence from participants' perspective, the role it plays in personal performance of a healthcare professional in their role, and the need to enhance intercultural competency skills and eliminate biases by indulging in continuing professional development.

2.1. Defining cultural competence [sub-theme]

This can be defined as the recognition and respect for diverse backgrounds, tailoring one's communication and care according to the person's background and culture, as well as eliminating any biases during the provision of care.

2.1.a. Respecting diverse backgrounds and provide tailored care

This refers to the recognising and respecting diverse backgrounds of persons and providing care without any discrimination.

2.1.b. Intercultural communication and cooperation

This refers to the ways in which a person communicate and collaborate with persons of different backgrounds and cultures in healthcare or academic settings.

**Codes**	**Respecting diverse backgrounds and provide tailored care**	**Intercultural communication and cooperation**
**Quotes**	Cultural competence to me, it means recognising that other people are different to yourself and they have different backgrounds, different identities, and being able to, uh, provide care for them regardless of their background in an equal manner. (P10)<br><br>You are able to understand the different cultures and respect different cultures. That you have the enough knowledge and confidence to act and	intercultural competence, just from the name sounds to me how well you can, uh, cooperate and communicate with different people from different cultures. It within your workplace or within educational institutions. (P11)<br><br>I think it's just to implement that different people have different opinions based off of their cultures and you should be able to, you know, work with people with these different backgrounds despite your different opinions. (P11)

	provide services, talk to people from different cultures without doing any harms as Socialized healthcare professional. (P11)<br><br>There are some people with different backgrounds and then you have to respect uh and then deal with their backgrounds and their beliefs and respect their opinions. (P9)	

2.2. Personal importance of cultural competence [sub-theme]

This theme refers to the realisation of the importance of cultural awareness and sensitivity, respecting and empathising with persons during diverse interactions. It also includes addressing cultural differences and language issues that may hinder achievement of a person-centred care.

2.1.a. Importance of cultural competence in interactions

This signifies the importance of being culturally aware, understand and respect the different backgrounds of individuals when interacting with them either in the capacity of a pharmacist or personal life. This also includes the ability to understand and empathise with people from different backgrounds and cultures, and being able to recognise and respect diverse perspectives during an interaction.

2.1.b. Application of cultural competence in interactions

Application of cultural competence skills means being culturally aware and sensitive when interacting with persons from different backgrounds. This further includes addressing language barriers and cultural differences in healthcare service delivery.

2.2.c. Importance in patient care

This recognises the role of cultural awareness and sensitivity during patient care and how they impact the achievement of a person-centred care.

Codes	Importance of cultural competence in interactions	Application of cultural competence in interactions	Importance in patient care

**Quotes**	So just like being able to understand how other people's culture like affects your interactions with them and like how important, like how embedded culture is. (P1)<br><br>just try and find out more about where other people come from cause it will be like important in trying to boost your relationships not only with your patients but with other people that you surround yourself with. (P1)<br><br>I think the lecture solidified how I felt about the importance of culture and in pharmacy. (P3)<br><br>I think it's important to have the ability to understand and interact effectively with people from different cultures. It allows us to have self-awareness, have empathy towards one another, and the chance to create long lasting relationship. (P3)<br><br>it is important to be aware of other people's ethnic, backgrounds, like not cause offence to them and like accidentally insulting them or something. But I think it's important for both Pharmacy and in just in life in general. (P8)<br><br>I realized that it's a really good thing to do interprofessional work specially as a healthcare where you work in a country which is multicultural country. So you are	I would say just make sure you know the language. Make sure you're empathetic and understanding, no matter what, and don't try and undermine someone's health condition just because maybe their English is not so good because you might be like missing it important red flag symptoms. (P10)<br><br>Probably maybe like how to deal with it. Like If you face any problems in the future look like in a cultural difference or requires a cultural competence, and like how to actually handle it or deal with it as like in a professional way, as a pharmacist, how would we deal with it in the future. if you actually face that in and that in a work setting. (P6)<br><br>Now more specific, too specific scenarios or like situations where I will be in, I just understand them more and I know how to deal with them. (P7)	I think the most important thing in the discussion was recognising that from there is a learning need for cultural competence. So we can provide care to patients. (P10)<br><br>Maybe just be like a little bit more cautious about what you say in front of patients. Just be like cautious what you say to different patients. different patients may react differently to information that you give them. (P8)<br><br>If you lack intercultural competence, so if you lacking intercultural competence, you could damage a patient's health and for that reason it's more important in pharmacy practice. (P10)<br><br>how we interact with people from other cultures has a direct impact on their healthcare that they receive. So, for that reason it's relevant and useful to know in pharmacy practice. (P10)<br><br>when it comes to pharmacy practice, it's just having a cultural competence is important because it helps you to achieve that patient centred care much better and better quality. (P11)

	able to understand people difference. You kind of have the duty to do it. (P11)<br><br>we need to actually know about culture as we can face it in our pharmacist journey in the future. (P6)		

2.3. Gaps and impact of cultural Competence in healthcare [sub-theme]

This sub-theme highlights the gaps in cultural awareness among most members of the public and some healthcare professionals. It emphasises the need to possess necessary skills to deliver effective healthcare and achieve positive patient outcomes. It further calls for healthcare professionals to challenge themselves to address cross-cultural issues in healthcare indulge in continuing professional development to improve their intercultural competency.

2.3.a. Gap in cultural awareness

This means that there an unfamiliarity with diverse cultures among most members of the public.

2.3.b. Importance of cultural awareness among healthcare professionals

This refers the essential knowledge and skills healthcare professionals must possess to be able to successfully deliver effective care in cross-cultural interactions.

2.3.c. Cultural Competence improves patient outcomes

This code highlights the importance of cultural competence skills namely, intercultural knowledge, communication and empathy, and their impact on patient outcomes.

2.3.d. Embracing cultural challenges

This means that the healthcare professionals have to challenge themselves to address cross-cultural issues. It further includes continuing professional development to self-identify and eliminate biases that could hinder care.

Codes	Gap in cultural awareness	Importance of cultural awareness among healthcare professionals	Cultural competence improves patient outcomes	Embracing cultural challenges
Quotes	I don't see many people kind of eager to find out about other people's culture in a sense. (P1)<br><br>Most of the people here at least, or at least I can say the people that I've been in contact with, or people that I've seen. They are not familiar with all the cultures apart from just some common cultures that you can see in the UK. (P2)<br><br>Some other healthcare professionals such as pharmacy dispenses and are rarely aware of the importance of intercultural competence. As I have witnessed in some situation where pharmacy dispenser gets confused, if patient behaved differently and because of a religion or their culture beliefs and they were expecting him or her to behave as normal, but that wasn't the case and then they were confused. (P9)<br><br>I think another gap is that is the communication between the pharmacist and patient because maybe the patient doesn't like to share the culture. You know, maybe they're shame of the culture. (P11)<br><br>I feel like the gap is like the knowledge of different culture. It is like you need to have that knowledge background about	I think that every pharmacist has to have a good knowledge of different religions and cultures. Because I think it makes the patient more able to communicate and then more open to speak up about their diseases or illnesses with health professional for better treatment. (P9)<br><br>I think that would kind of like affect a future pharmacist and practice if they're not eager to find out where their patients are coming from and trying like give a better, like better healthcare towards them because like surrounding their culture, I think that that's why it's quite important. (P1)<br><br>I think that pharmacist and healthcare professionals in general should familiarise themselves with diverse cultural backgrounds because it will affect the care that they give. This includes different beliefs, different values and healthcare practices, and how this will affect their response to medication and treatment. (P3)	Being interculturally competent improves patient outcomes because it allows you to communicate better and be more empathetic, which has an impact on the patients quality of care. (P10)<br><br>We need to have the knowledge, uh of different background of the patient because it helps in offering a patient centred care. (P9)<br><br>I think if they try to reach that communication to be able to communicate the feelings or the cultural background, then the pharmacist might get a better understanding of what's going on. (P11)	But this is something I would want to just like try and like step out of your comfort zone. (P1)<br><br>I think one of the gaps is being able to received training for putting aside your different political beliefs. Maybe I might have a different political belief to you, but we still have to be able to provide that same care and we haven't really been given that training before. (P10)

	each culture to deal with each situation. And I feel like not everyone has that knowledge about people's backgrounds. And if you don't have it, it's going to be a bit hard to deal with the situation and it's better to actually have the knowledge. (P6)<br><br>I think a gap would be the lack of knowledge of different cultures. (P11)			

3. Student-Preferred Pedagogy [Theme]

This theme includes the learning and assessment methods that students preferred for teaching and assessing cultural competence in the curriculum. It includes students' preference for integrating interactive and engaging learning methods such as workshops and placements, and several assessments techniques such as scenario-based, simulation-based, placement-based, and structured assessments.

3.1. Preferred learning methods [sub-theme]

This sub-theme mentions the preference of students to incorporate learning methods in the curriculum that focus on student engagement, are interactive, and help students acquaint themselves with cross-cultural situations arising in real-world healthcare settings.

3.1.a. Interactive workshops

This refers to integrating learning methods which are interactive and allow for roleplays and discussion such as workshops, to improve students' understanding of cross-cultural scenarios, requiring demonstration of cultural competence.

3.1.b. Practical experience

This refers to integrating direct, hands-on, experiential learning opportunities such as placements that provide exposure to cross-cultural situations in healthcare settings.

**Codes**	**Interactive workshops**	**Practical experience**
**Quotes**	Maybe workshops and patient interactions, I think that would be good. (P1)<br><br>I think maybe have a bit more open discussion about different people's experiences. I think part of the whole content is like understanding everyone's different and since we are such diverse course people international from all over the world and home students as well, it would be a bit more relatable, If the students were involved more in like a discussion. (P4)<br><br>It's a good idea to have it in interactive workshops. (P11)<br><br>I think the best way is with interactive workshops. (P11)<br><br>In the workshops you could have mock role plays, so you could like someone can be acting as a patient, someone acting as a pharmacist. (P10)<br><br>I think I would benefit from workshops that would allow me to build on skills such as like analysing and being able to interpret verbal and nonverbal cues with patients who may not speak English. (P3)	Maybe talk in practice like doing in practical's like simulations instead of lecture. (P7)<br><br>I would recommend that practical experience for students. (P9)<br><br>So like in placements, it is a good way to actually get direct experience with other patients from different cultures. (P10)

	The content if delivered as a lecture won't be as effective as if it was as a workshop because students could be more interactive with the content, and it could be like scenario based. (P4)<br><br>Maybe a workshop would be good in which they could explain some certain different factors from different parts of the world. (P5)<br><br>I feel like one lecture is not enough to explain everything and show everything. probably better to have like more case studies and like a workshops and stuff. (P6)<br><br>Maybe like workshops or something. (P8)	

3.2. Preferred assessment methods [sub-theme]

This includes the myriad techniques students would prefer to be assessed by for their intercultural competence skills. These include placement-based, scenario-based, structured, and simulation-based assessments.

3.2.a. Placement-based assessments

Placement-based assessment refers to assessment of cultural competence skills during an experiential learning activity such as placements.

3.2.b. Scenario-based and structured assessments

This includes specific evaluation techniques and assessment methods to measure a student's cultural competence skills in an examination. This includes multiple choice questions and scenario-based questions.

3.2.c. Simulation-based assessments

It refers to employing mock roleplays and objective structured clinical examinations (OSCEs) to assess students' intercultural competencies.

Codes	Placement-based assessments	Scenario-based and structured assessments	Simulation-based assessments
**Quotes**	I think intercultural competence is something you have to have in general as a professional, so it's maybe like part of professional practice. like scenario-based decision making. (P4).<br><br>I think it says that placement activity assessment was an example. I think that would be a great way because maybe we can like role play different scenarios with patients of different backgrounds. It allows us to be practical and put ourselves in their shoes. (P3)	Maybe an MCQ such as<br>What would you do in this case? Uh, like more of a ethical problem, I guess it would be, right?<br>Or maybe like short answer question, or you can even make maybe a long answer question. You know, like a situation with a patient like that maybe. So it's like making a scenario and see how they will react to it. (P5)<br><br>Culture competence can be assessed as a group study. Group case study like presenting a problem or try to solve the problems. (P6)<br><br>Probably MCQ like oriels. what is the most professional thing you can do in a scenario? What's the best option and what's the least best option in the certain scenario and you<br>just rank your answers. (P10)<br><br>I think it would fit more in the scenario-based questions. (P4)<br><br>If we are going to be assessed on it in Exams, probably a multiple-choice	I feel like if it's more of a simulation thing, like in our OSCE, you can do like ohh, I'm a person of color or an Asian person and I have different beliefs from you. What do you do then? So that will put you in a difficult position to actually deal with the problems. I feel like it's a thing for OSCE mostly like put it in the OSCE; don't think it fits in exam questions. (P7)<br><br>I would say it's quite according because I don't know how, like kind of add some aspects in there and that can be like sort of marks or something and it for example if you had somebody who was a of a certain religious background or some a cold like a certain diet and like the question was like what kind of like are you going to give them like a capsule or something that has gelatine in it that's like kind of like a consideration can make. So I think OSCE might be might be good for the assessment. (P1)<br><br>in Mpharm I suppose it's one way to practice your intercultural competences Is by having an actual organized practice sessions with the teacher. For example, how we prepare for consultations exam, OSCE and all of the stuff where we had someone examine us. They should also do similar for interculture competence as well. (P11)

		question rather than long answer question. (P8)	You know how Pam Sessions were in the first year where they were actors, they put you in the situation to give the consultation to the patient. So I think the same way you can like actors from different cultures, who knows what's going on and then see how the student would react to different cultures if you, if you, if you really want to teach the competency and stuff, I think that that's the best way. If you want to assess the students put the students in this situation, to see how they act. I think it should be assessed as practical and activity based. (P11)<br><br>I think we have OSEC's ahead of us and we have some practical's as well that we talk to the patients. I think the best assessment could be dealing as practice… And the teachers can monitors as if we consider the patients culture. (P2)
